# Ototoxicity of a Single Fulminant Episode of Acute Otitis Media in Children: A Long-Term Follow-Up

**DOI:** 10.3390/audiolres16030093

**Published:** 2026-06-22

**Authors:** Matija Švagan

**Affiliations:** Department of Otolaryngology, Head and Neck Surgery, University Medical Center, 2000 Maribor, Slovenia; matija.svagan@ukc-mb.si

**Keywords:** acute mastoiditis, distortion-product otoacoustic emissions, extended high-frequency audiometry, mastoidectomy, sensorineural hearing loss, vestibulo-ocular reflex

## Abstract

**Background/Objectives:** Recurrent acute otitis media (AOM) in children is known to cause cumulative cochlear and vestibular injury. Whether a single fulminant episode severe enough to require surgical intervention produces an analogous long-term audiovestibular signature, and whether infection severity contributes to outcome independently of cumulative episode count, is unclear. The present study addressed this gap. **Methods:** In this single-centre retrospective cohort study, 65 paediatric patients who had undergone surgical treatment for acute mastoiditis—the fulminant form of AOM—between July 2001 and March 2021 were assessed a median of 11.5 years after surgery. Of these, 35 had undergone mastoidectomy with tympanostomy and 30 had undergone tympanostomy alone because their episode had not been severe enough to require mastoidectomy. Thirty-two age-matched healthy volunteers (one ear each) formed the control group, yielding 97 ears in three groups (Group TM, 35 ears; Group T, 30 ears; Group C, 32 ears). Extended high-frequency pure-tone audiometry (125–20 kHz), distortion-product otoacoustic emissions (DPOAEs), single-frequency and wideband tympanometry, ipsilateral acoustic reflex thresholds, and lateral-canal vestibulo-ocular reflex gain were measured. **Results:** Both operated groups showed significantly elevated audiometric thresholds in the high- and extended high-frequency ranges compared with controls (HTA: χ^2^ = 24.25, *p* < 0.001), with corresponding reductions in DPOAE amplitudes (HTA: χ^2^ = 25.04, *p* < 0.001). Group TM did not differ significantly from Group T at any frequency band, indicating a negligible additional contribution of mastoidectomy itself. Acoustic reflex thresholds were elevated in Group TM. Vestibulo-ocular reflex gain was within reference ranges in all groups. **Conclusions:** A single fulminant episode of acute middle-ear infection in childhood—whether severe enough to require mastoidectomy or treated by tympanostomy alone—was associated, more than a decade later, with significantly elevated audiometric thresholds closely resembling those reported after multiple recurrent infections, supporting an effect of infection severity independent of cumulative episode count. Long-term audiological follow-up with extended high-frequency audiometry and otoacoustic emission testing is warranted, irrespective of whether mastoidectomy was required.

## 1. Introduction

Acute otitis media (AOM) is among the most prevalent diseases of early childhood. A substantial body of evidence has established that recurrent middle-ear infections can exert ototoxic effects on the inner ear. The pathophysiological premise common to most of the literature is that middle-ear inflammation increases the permeability of the round window membrane, permitting bacterial endotoxins and exotoxins, pro-inflammatory cytokines, and reactive oxygen species to diffuse into the perilymph. The resulting injury comprises serous (sterile) labyrinthitis, hair-cell damage—particularly to the outer hair cells of the basal turn of the cochlea—and disruption of the blood–labyrinth barrier. The same diffusion pathway exposes the vestibular end-organs (saccule, utricle, and semicircular canals), which is why vestibular and balance disturbances are increasingly recognised as parallel sequelae rather than incidental observations [1,2,3,4].

The available data consistently demonstrate that high-frequency, and, in particular, extended high-frequency, sensorineural hearing loss is over-represented in children with recurrent AOM compared with controls [5,6,7,8,9]. By contrast, vestibular involvement in paediatric AOM remains clinically under-recognised and incompletely characterised. More evidence is available for otitis media with effusion (OME), in which approximately one-third of affected children exhibit measurable vestibular dysfunction. The toxin-diffusion mechanism implicated in cochlear hair-cell injury is presumed to operate analogously on the vestibular hair cells [10,11,12,13,14].

A notable gap in the current literature concerns the long-term, disease-specific consequences of a single fulminant episode of AOM severe enough to necessitate surgical intervention—tympanostomy, mastoidectomy, or both. The present study was undertaken to address this gap. It is also well established that the surgical procedures themselves—tympanostomy and mastoidectomy—do not appear to confer any major adverse effect on hearing or vestibular function [15].

## 2. Materials and Methods

Sixty-five children who had been surgically treated for acute mastoiditis (AM)—the fulminant manifestation of AOM—at the Department of Otorhinolaryngology, Head and Neck Surgery, University Medical Centre Maribor, between July 2001 and March 2021, were invited to participate. Surgical management had comprised cortical mastoidectomy with concurrent tympanostomy in 35 patients, who were assigned to Group TM, and tympanostomy alone in 30 patients in whom the fulminant episode had not advanced to a degree requiring mastoid drainage, who were assigned to Group T. The 30 tympanostomy-only patients were identified consecutively from the departmental database and matched, at the group level by median age, to the mastoidectomy cohort. Patients in whom the operated ear subsequently experienced more than one non-fulminant episode of AOM or developed any additional complication of the underlying acute mastoiditis, were excluded from either surgical group. Surgical indications during the recruitment period followed established clinical practice [16]. A separate cohort of 32 healthy volunteers, each free of any documented or self-reported history of middle-ear infection, hearing loss, or vestibular complaint, and group-matched to the operated cohorts by median age, served as the control group (Group C). In control participants, one ear was randomly selected for analysis, while in the operated groups, only the operated ear was assessed.

Audiological, otoacoustic, immittance, and vestibular assessments were carried out between 1 September 2023 and 15 May 2026. The final dataset comprised 97 ears (Group TM, 35; Group T, 30; Group C, 32).

The greater part of the mastoidectomy cohort (30 of 35 patients in Group TM) and the entire healthy control cohort (Group C) of the present study are drawn from the same institutional database as the patients previously reported by Švagan (2025) [17]; the remaining five mastoidectomy patients have been newly recruited for the present work and underwent identical audiovestibular testing. The earlier publication examined long-term morphological and ventilatory outcomes of the mastoid compartment—pneumatisation patterns, residual cavity characteristics, and related mastoid morbidity. The present study differs from it principally in the research question. The earlier work examined the structural and functional sequelae of mastoidectomy; the present work addresses the audiovestibular sequelae of a single underlying fulminant infection and asks whether the severity of an individual episode contributes to long-term inner-ear outcome independently of the cumulative episode count established in the recurrent-AOM literature. Additionally, the design has been extended. A third cohort of tympanostomy-only patients (Group T), absent from the previous publication, has been added; the inclusion of this group allows the contribution of mastoidectomy as a surgical procedure to be distinguished from the contribution of the underlying inflammatory insult to the long-term audiovestibular outcome.

The audiological assessment protocol and the instrumentation employed in the present study were identical to those previously described by the author [17], thereby ensuring methodological continuity between the two investigations. Extended high-frequency pure-tone audiometry (125–20 kHz) was performed using an Interacoustics AC40 clinical audiometer (Interacoustics, Denmark). The AC40 audiometer’s reliable minimum output at the test frequencies used was 10 dB HL, and intervals below this floor cannot be presented with adequate calibration tolerance. For these reasons, and to remain conservative in classifying participants with no clinically meaningful hearing loss, the upper limit of normal hearing was set at 10 dB. To facilitate statistical comparison, audiometric thresholds were grouped into a low-tone average (LTA, <500 Hz), middle-tone average (MTA, 0.5–4 kHz), high-tone average (HTA, 4–8 kHz), and extended high-tone average (EHTA, 8–20 kHz). The MTA was calculated using the same procedure as the conventional pure-tone average (PTA) [18]. Single-frequency (226 Hz) tympanometry and stapedial reflex thresholds (at 500, 1000, 2000, and 4000 Hz) were obtained using an Interacoustics AT235 tympanometer (Interacoustics, Middelfart, Denmark). Wideband tympanometry was performed using Interacoustics Titan hardware (Interacoustics, Denmark), recording external auditory canal volume, tympanometric peak pressure (TPP), compliance, and middle-ear resonant frequency. Distortion-product otoacoustic emissions (DPOAEs) were acquired with Interacoustics Eclipse equipment (Interacoustics, Denmark) using stimulus levels of 65 dB SPL (f1) and 55 dB SPL (f2), an f2/f1 ratio of 1.22, a test duration of 60 s, and a minimum reliability criterion of 98%. Measurements were performed in duplicate across frequencies from 500 Hz to 10 kHz; only emissions meeting the reliability criterion were retained, after which values were averaged and then frequency-averaged using the same banding scheme as the audiometric data. Vestibular function was assessed by measuring the response of the vestibulo-ocular reflex (VOR) elicited from the lateral semicircular canal, this being the most exposed canal during acute middle-ear inflammation. Recordings were obtained with a Natus ICS Impulse system (Natus, Middleton, WI, USA), with a minimum of 30 repetitions per side to ensure replicability.

All statistical analyses were performed using Jamovi version 2.7.26.0. Descriptive statistics were used to characterise the sample. The Shapiro–Wilk test was applied to assess the distribution of continuous variables; because the data were not normally distributed, continuous results are reported as medians with the corresponding interquartile range (Q1, Q3), and categorical results as frequencies and percentages. Mean values are reported where appropriate. Between-group comparisons were performed using two-sided Pearson’s χ^2^ tests, the Kruskal–Wallis test, and the Mann–Whitney U test. For Kruskal–Wallis comparisons, epsilon-squared (ε^2^) was calculated as the effect size, with ε^2^ ≥ 0.08 interpreted as a moderate effect and ε^2^ ≥ 0.26 as a large effect. Pairwise post hoc comparisons following a significant Kruskal–Wallis test were carried out using the Dwass–Steel–Critchlow–Fligner procedure. Associations between continuous variables were assessed with Kendall’s rank correlation coefficient (τ_b). A *p*-value of less than 0.05 was considered statistically significant.

There are some limitations of this study. The retrospective, single-centre design of this study introduces potential sources of bias. Participation in the audiovestibular assessment was voluntary; willingness and ability to attend the study could introduce a bias in either direction. The cohort also comprises individuals at different developmental stages at the time of testing, which may affect the comparability of audiometric thresholds across participants. The audiometric cut-off used to define normal hearing (10 dB HL) introduces a floor effect—most apparent in Group C, in which 32 of 32 ears were truncated at 10 dB HL in the HTA. This may inflate apparent between-group differences in the low- and middle-tone averages, although it does not affect the high- and extended high-frequency findings on which the principal conclusions depend, since those bands lie well above the floor.

## 3. Results

A single episode of uncomplicated AOM preceding AM was documented in 6.2% (n = 4) of patients preoperatively and in 4.6% (n = 3) postoperatively. No patient experienced more than one additional uncomplicated episode, yielding a maximum of one non-fulminant AOM in addition to the surgical episode. The remaining patients had no documented history of ear infections. Neither the patients nor their parents reported any pre-existing hearing loss prior to the onset of the fulminant infection, and 98.5% (n = 64) had successfully passed routine neonatal hearing screening. Furthermore, there were no auto- or hetero-anamnestic data suggesting that patients in the operated groups had experienced any additional risk factors or conditions that could account for high-frequency hearing loss following surgery. The median age at surgery was 2.16 years (Q1: 1.42; Q3: 4.08), with the youngest patient aged 0.64 years and the oldest 11.33 years. The left ear was operated upon in 58.5% (n = 38) of cases, and 60% (n = 39) of patients were male. The median interval from symptom onset to surgery was 3.77 days (Q1: 1.71; Q3: 6.78). At the time of testing all operated ears had intact, well-positioned tympanic membranes.

The median leukocyte count was 15.2 × 10^9^/L (Q1: 12.3; Q3: 20.01; range 1.5–35.2 × 10^9^/L). The median C-reactive protein concentration was 96.0 mg/L (Q1: 54.5; Q3: 153.0; range 5–280 mg/L). *Streptococcus pneumoniae* was the predominant pathogen, isolated in 56.9% (n = 37) of cases. *Streptococcus pyogenes* (group A streptococcus) was identified in 20.0% (n = 13), *Haemophilus influenzae* in 9.2% (n = 6), while *Turicella otitidis*, *Pseudomonas aeruginosa*, and *Staphylococcus aureus* were each isolated in 1.5% (n = 1) of cases. Cultures were sterile in 9.2% (n = 6). Systemic antibiotics, either in oral or intravenous form, were administered prior to surgery in 36.9% (n = 24) of patients—43.3% (n = 13) in Group T and 31.4% (n = 11) in Group TM. The most frequently prescribed antibiotic was amoxicillin-clavulanate, accounting for 60% of all prescriptions (30.8% in Group T and 29.2% in Group TM), followed by cefotaxime (13.9%), cefuroxime (12.3%), ceftriaxone (9.2%), and ceftazidime and cefepime each representing 1.5% per group. Postoperatively, all patients received intravenous antibiotics for a minimum of 7 days; however, data on the total duration of antibiotic treatment are incomplete, as therapy was continued at outside institutions.

The interval between surgery and assessment in the present study was a median of 11.5 years (Q1: 7.4; Q3: 13.6; range 5.1–22.1 years). The overall median age at the time of testing was 14.2 years (Q1: 11.2; Q3: 16.3; range 6.63–24.8 years); the median age was 14.0 years (Q1: 11.0; Q3: 15.5) in Group TM, 14.1 years (Q1: 11.00; Q3: 15.4) in Group T, and 14.5 years (Q1: 13.1; Q3: 19.5) in Group C.

### 3.1. Extended High-Frequency Pure-Tone Audiometry

**Figure 1:**
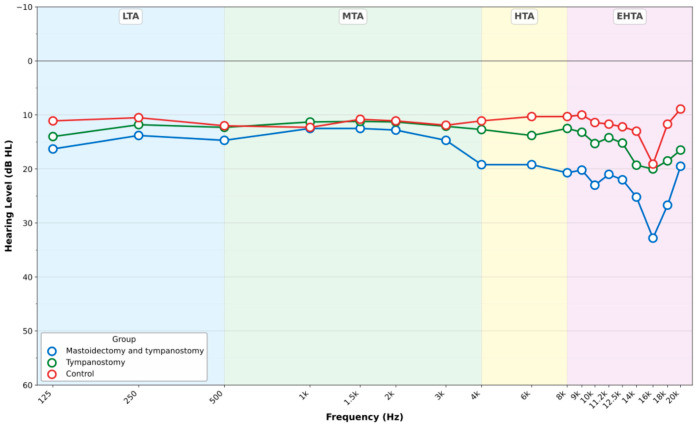
Mean audiometric thresholds for all three groups, presented as a clinical audiogram; LTA, low-tone average (<500 Hz); MTA, middle-tone average (0.5–4 kHz); HTA, high-tone average (4–8 kHz); EHTA, extended high-tone average (8–20 kHz).

Audiometric thresholds by frequency band and group are presented in Table 1; the corresponding clinical audiogram is shown in Figure 1.

Statistically significant between-group differences in hearing thresholds were identified across all three groups for the LTA (χ^2^ = 9.91, *p* = 0.007, ε^2^ = 0.103), MTA (χ^2^ = 6.06, *p* = 0.048, ε^2^ = 0.063), HTA (χ^2^ = 24.25, *p* < 0.001, ε^2^ = 0.256), and EHTA (χ^2^ = 6.48, *p* = 0.033, ε^2^ = 0.071). Pairwise comparisons revealed no significant differences between Groups TM and T at any frequency average. A significant difference between Groups T and C was found only for the HTA (W = 4.18, *p* = 0.009). Group TM differed significantly from Group C in the LTA (W = 4.4, *p* = 0.005), HTA (W = 6.88, *p* < 0.001), and EHTA (W = 3.49, *p* = 0.036), but not in the MTA (W = 2.58, *p* = 0.162). A small number of Group TM ears (n = 2) showed threshold elevations greater than 60 dB HL in the high-frequency or extended high-frequency range, indicating that severe sensorineural hearing loss can result from a single fulminant episode.

Age at the time of infection was significantly correlated with higher thresholds in the EHTA (τ_b = 0.239, *p* = 0.007). By contrast, no significant correlation was found with the interval between the infection and the present assessment. No association was observed between poorer hearing thresholds and the bacterial pathogen isolated, the leucocyte count, the C-reactive protein concentration, or antibiotic usage.

### 3.2. Distortion Product Otoacoustic Emissions

**Figure 2:**
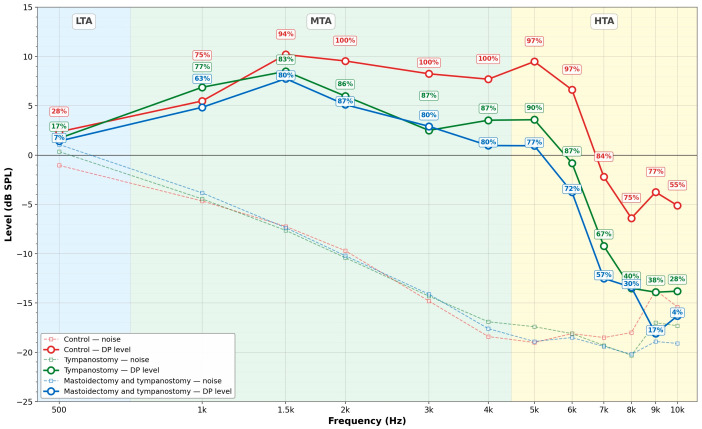
Mean DPOAE for all three tested groups presented in the form of a DP-gram. Values above each point represent detection ratio; LTA, low-tone average (500 Hz); MTA, middle-tone average (1–4 kHz); HTA, high-tone average (5–10 kHz).

Noise-corrected DPOAE levels, frequency-averaged in a manner analogous to the audiometric data—low-tone average (LTA; 500 Hz), middle-tone average (MTA; 1–4 kHz), and high-tone average (HTA; 5–10 kHz)—are presented in Table 2 and displayed as a DP-gram in Figure 2. Because DPOAE acquisition was limited to 500–10 kHz, the band boundaries were adjusted accordingly. The LTA was subsequently excluded from inferential analysis because of unreliable response detection at this frequency (Figure 2). Noise-corrected DPOAE values differed significantly between groups for the HTA (χ^2^ = 25.04, *p* < 0.001, ε^2^ = 0.261), but not for the MTA (χ^2^ = 5.25, *p* = 0.081, ε^2^ = 0.055). Pairwise comparisons revealed no significant differences between Groups TM and T at any frequency band. Group T differed significantly from Group C in the HTA (W = 4.71, *p* = 0.002) but not in the MTA. Group TM likewise differed significantly from Group C in the HTA (W = 6.94, *p* < 0.001) but not in the MTA. DPOAE levels in the MTA (τ_b = −0.486, *p* < 0.001) and HTA (τ_b = −0.461, *p* < 0.001) correlated significantly with audiometric thresholds at the corresponding frequency bands.

### 3.3. Middle Ear Impedance Testing

Single-frequency and wideband tympanometry showed that Group C had significantly larger external auditory canal volumes than either Group TM or Group T (χ^2^ = 21.45, *p* < 0.001, ε^2^ = 0.223). No other tympanometric measure—TPP or compliance at 226 Hz or 1000 Hz—differed significantly between groups. The median resonant frequency was 754 Hz in Group C, 722 Hz in Group T, and 668 Hz in Group TM; no significant intergroup differences (χ^2^ = 2.63, *p* = 0.268, ε^2^ = 0.034) were detected.

Ipsilateral acoustic reflex thresholds differed significantly between groups at all tested frequencies: 500 Hz (χ^2^ = 9.49, *p* = 0.003, ε^2^ = 0.098), 1 kHz (χ^2^ = 19.62, *p* < 0.001, ε^2^ = 0.204), 2 kHz (χ^2^ = 15.87, *p* < 0.001, ε^2^ = 0.165), and 4 kHz (χ^2^ = 18.69, *p* < 0.001, ε^2^ = 0.194). Pairwise comparisons revealed that Group TM differed significantly from both Group C and Group T at all four frequencies, whereas Groups C and T did not differ significantly at any frequency.

### 3.4. VOR Measurements

The overall median VOR gain was 0.88 (Q1: 0.81; Q3: 0.95). Per-group medians were 0.89 (Q1: 0.81; Q3: 0.96) in Group C, 0.86 (Q1: 0.81; Q3: 0.92) in Group T, and 0.88 (Q1: 0.80; Q3: 0.95) in Group TM. No significant differences were found between the Groups concerning VOR gain (χ^2^ = 0.71, *p* = 0.700, ε^2^ = 0.007), variability of VOR gain measurements (χ^2^ = 0.002, *p* = 0.984, ε^2^ = 0.003), number of overt (χ^2^ = 0.482, *p* = 0.791, ε^2^ = 0.005), and covert (χ^2^ = 0.32, *p* = 0.849, ε^2^ = 0.003) saccades.

## 4. Discussion

The aim of the present study was to assess the ototoxic effects of a single, fulminant episode of acute middle-ear infection. Earlier work has demonstrated that recurrent non-fulminant AOM exerts a cumulatively deleterious effect on hearing, with the magnitude of impairment increasing in proportion to the number of repeated infections [19]. From a pathophysiological standpoint, the proposed mechanism of inner-ear injury—increased permeability of the round window membrane, direct damage to cochlear hair cells by bacterial toxins and inflammatory mediators, and, possibly, macrophage-mediated hair-cell loss—is likely to operate in much the same way in a single fulminant episode as in repeated non-fulminant infections [20,21,22,23,24,25]. It remains uncertain, however, whether the severity of an individual infection contributes to the eventual degree of hearing loss, or whether the cumulative number of episodes is the dominant determinant.

Hearing-threshold elevation was most pronounced in the high-frequency and extended high-frequency ranges, in keeping with the existing literature [6,7,8,9,19]. Median audiometric thresholds indicated a minor hearing loss (of the order of 10 dB) across all frequencies in both post-infection groups compared with controls. Detailed audiometric analysis revealed statistically significant differences between each test group and the control group, predominantly in the high-frequency and extended high-frequency ranges. Notably, no statistically significant differences in frequency-band averages were observed between Groups T and TM, indicating that, at the group level, audiometric outcomes did not differ depending on whether mastoidectomy was performed in addition to tympanostomy. By contrast, direct comparison of Group TM—corresponding to the more severe, mastoidectomy-requiring infection—with the control group revealed significantly elevated hearing thresholds in all frequency averages except the MTA. This pattern is consistent with a more severe and prolonged inflammatory insult in the ears that ultimately underwent mastoidectomy, with a correspondingly greater risk of inner-ear damage. Furthermore, when the sloping configuration of the pure-tone audiogram in our mastoidectomy group is compared with that reported in the literature, it broadly resembles the pattern observed after multiple (>8) recurrent infections [17], although the effect is less pronounced at the highest frequencies. A comparable dose–response relationship has been reported in children with recurrent secretory otitis media, in whom four or more episodes were sufficient to produce a statistically significant elevation of extended high-frequency thresholds [26]; the present finding suggests that a single fulminant episode may produce an analogous, though slightly attenuated, audiometric signature. In the mastoidectomy group, acoustic trauma during surgery remains a recognised potential risk, principally on account of the high-intensity noise generated by high-speed drilling, which is transmitted to the inner ear by bone conduction and may affect high-frequency auditory sensitivity. Furthermore, the available evidence suggests that the threshold shift induced by drill noise is largely reversible [27,28,29,30,31]. Nevertheless, the comparable audiometric patterns observed in Groups T and TM support the interpretation that inflammatory processes, rather than surgical intervention, are the predominant contributor to the observed auditory deficits. The present data do not reveal a detectable additional contribution of mastoidectomy to long-term hearing outcomes at the group level, a finding consistent with a previous study employing a within-subject paired design, though this null finding should be interpreted with appropriate caution [17].

Although the group-level threshold elevation was modest, within-group variability was substantial: two Group TM ears showed severe-to-profound high-frequency loss, demonstrating that a single fulminant episode can occasionally produce clinically significant cochlear damage, most likely on the basis of serous labyrinthitis. These two extreme values introduced a small upward skew into the group means, although the medians used for inferential comparison were largely unaffected.

It is important to note that neither test group differed from the control group in the MTA, which is calculated in the same manner as the widely reported pure-tone average (PTA) [18]. Consequently, reporting the PTA alone would have masked the audiometric differences between the control and test groups.

The audiometric findings—similarity between the two surgical groups, with poorer performance in both relative to controls—were corroborated by the DPOAE data, which showed a parallel pattern. DPOAE amplitudes in the HTA were significantly lower in both Group T and Group TM than in the control group, whereas no significant difference was found in the MTA. Direct comparison between Groups T and TM yielded no significant difference at any frequency band.

Acoustic immittance testing revealed elevated acoustic reflex thresholds in Group TM, indicative of altered middle-ear mechanics. This interpretation is reinforced by the lower—though non-significant—median resonant frequency in the same group, a pattern consistent with a mass-loading effect from postoperative scarring [32]. A primarily cochlear origin is unlikely at the reflex frequencies tested (500–4000 Hz), as DPOAE amplitudes in the corresponding MTA band did not differ significantly between groups; a retrocochlear contribution cannot be formally excluded in the absence of electrophysiological testing (such as auditory brainstem response, ABR) in the present protocol, but is improbable given the preserved VOR gain and the absence of neurological history in this cohort. The apparent dissociation between elevated acoustic reflex thresholds in Group TM and the otherwise unremarkable tympanometric findings merits comment. Single-frequency tympanometry and its associated compliance measure reflect the passive mechanical movement of the tympanic membrane and ossicular chain under quasi-static pressure changes, whereas the acoustic reflex involves active, dynamic muscular contraction. Subtle structural changes—such as scarring around the stapes or stapedial tendon—could elevate the reflex threshold without producing a measurable change in static compliance. The reduced external auditory canal volume documented in the operated groups would also be expected to attenuate the effective stimulus delivered to the cochlea by a small but non-trivial amount, raising the elicitation threshold of the reflex without altering passive tympanometric measures. A statistically significant reduction in external auditory canal volume was also observed in Groups T and TM compared with controls; the underlying cause remains uncertain. Possible explanations include postoperative scarring of the external auditory canal and altered temporal bone pneumatization following severe infection [33]. The clinical significance of this finding remains undetermined, and its interpretation as an objective indicator of middle-ear status should be approached with caution.

All VOR gain values lay within the published reference ranges [34]. No significant differences were found between groups in VOR gain, in the trial-to-trial variability of VOR gain measurements, or in the detection of compensatory saccades. Taken together, these findings indicate that, under the present testing protocol, no detectable alteration in lateral semicircular canal function was demonstrable in the previously infected ears. The vestibular protocol in the present study was confined to high-frequency, high-acceleration testing of the lateral semicircular canal via the video head impulse test. The vertical canals and the otolithic end-organs were not tested, and low-frequency stimulation of the lateral semicircular canal had not been performed. Subtle dysfunction within these vestibular subsystems cannot, therefore, be excluded, and a fuller test battery in a future prospective study would clarify whether the negative vHIT finding extends to the entire vestibular periphery.

## 5. Conclusions

A single fulminant episode of acute middle-ear infection in childhood—whether severe enough to require mastoidectomy or treated by tympanostomy alone—was associated, more than a decade later, with significantly elevated audiometric thresholds and reduced distortion-product otoacoustic emissions, particularly in the high-frequency and extended high-frequency ranges. The comparable audiometric and DPOAE patterns observed in the tympanostomy-only group and the mastoidectomy-and-tympanostomy group suggest that the inflammatory insult appears to be the principal determinant of inner-ear injury, as no detectable additional contribution of mastoidectomy itself was identified at the group level.

The greater threshold elevations observed in the mastoidectomy group are consistent with a more severe and prolonged inflammatory process in those ears. Notably, the audiometric configuration after a single fulminant episode broadly resembles that previously described after multiple recurrent infections, suggesting that infection severity, in addition to the cumulative number of episodes, may contribute to long-term hearing outcome—a hypothesis that warrants prospective testing. Middle-ear impedance findings are compatible with subclinical residual middle-ear remodelling. In contrast, vestibulo-ocular reflex gain testing revealed no detectable abnormality of lateral semicircular canal function, but subtle dysfunction in other vestibular subsystems cannot be excluded. Taken together, these findings argue for long-term audiological follow-up—including extended-high-frequency audiometry and otoacoustic emission testing—in children who have sustained even a single fulminant episode of AOM, irrespective of whether mastoidectomy was required, and for prospective investigation of vestibular function with a fuller test battery in this population.

## Figures and Tables

**Table 1:** Audiometric thresholds by frequency band and group. Values are in dB HL. Statistical comparison by Kruskal–Wallis test with epsilon-squared (ε^2^) effect size. LTA, low-tone average (<500 Hz); MTA, middle-tone average (0.5–4 kHz); HTA, high-tone average (4–8 kHz); EHTA, extended high-tone average (8–20 kHz); IQR, interquartile range (Q3 − Q1). Audiometric thresholds were truncated at 10 dB HL because of the audiometer’s resolution at low intensities.

Band	Statistic	Group TM (n = 35)	Group T (n = 30)	Group C (n = 32)	χ^2^	*p*	ε^2^
**LTA**	**Median (IQR)**	**13.3 (7.5)**	**11.7 (3.3)**	**10.0 (1.7)**	**9.91**	**0.007**	**0.103**
	Mean	14.7	12.7	11.2			
	Min–Max	10.0–31.7	10.0–25.0	10.0–16.7			
**MTA**	**Median (IQR)**	**11.0 (5.0)**	**10.0 (2.0)**	**10.5 (2.3)**	**6.06**	**0.048**	**0.063**
	Mean	14.5	11.7	11.4			
	Min–Max	10.0–35.0	10.0–21.8	10.0–19.0			
**HTA**	**Median (IQR)**	**15.0 (8.3)**	**10.0 (3.3)**	**10.0 (0.0)**	**24.25**	**<0.001**	**0.256**
	Mean	21.7	13.2	10.2			
	Min–Max	10.0–108.0	10.0–30.0	10.0–13.3			
**EHTA**	**Median (IQR)**	**17.1 (14.6)**	**12.5 (7.5)**	**10.7 (4.3)**	**6.48**	**0.033**	**0.071**
	Mean	25.4	17.0	12.6			
	Min–Max	8.6–120.0	9.3–43.6	7.1–25.0			

**Table 2:** Noise-corrected distortion-product otoacoustic emission (DPOAE) levels by frequency band and group. Values are in dB SPL. Statistical comparison by Kruskal–Wallis test with epsilon-squared (ε^2^) effect size. LTA, low-tone average (500 Hz); MTA, middle-tone average (1–4 kHz); HTA, high-tone average (5–10 kHz); IQR, interquartile range (Q3 − Q1). † LTA values are presented for completeness only; unreliable response detection at this frequency precluded inferential analysis.

Band	Statistic	Group TM (n = 35)	Group T (n = 30)	Group C (n = 32)	χ^2^	*p*	ε^2^
**LTA †**	**Median (IQR)**	**4.7 (7.8)**	**4.4 (7.4)**	**6.3 (5.2)**	**—**	**—**	**—**
	Mean	4.3	6.3	6.8			
	Min–Max	−6.1 to 16.1	−4.5 to 17.4	−3.1 to 17.1			
**MTA**	**Median (IQR)**	**16.3 (14.3)**	**18.0 (10.9)**	**19.9 (8.9)**	**5.25**	**0.083**	**0.055**
	Mean	16.6	17.4	21.5			
	Min–Max	−0.8 to 32.5	−7.3 to 32.5	10.4 to 34.0			
**HTA**	**Median (IQR)**	**8.1 (6.6)**	**10.1 (10.5)**	**17.4 (6.0)**	**25.04**	**<0.001**	**0.261**
	Mean	8.1	10.6	17.1			
	Min–Max	−2.6 to 22.4	−1.1 to 23.9	1.8 to 30.0			

## Data Availability

The data presented in this study are available on request from the corresponding author due to privacy issues.

## Abbreviations

The following abbreviations are used in this manuscript:

AM	Acute mastoiditis
AOM	Acute otitis media
DPOAE	Distortion-product otoacoustic emissions
EHTA	Extended high-tone average
HTA	High-tone average
HL	Hearing loss
LTA	Low-tone average
MTA	Middle-tone average
OME	Otitis media with effusion
PTA	Pure-tone average
rAOM	Recurrent acute otitis media
TPP	Tympanometric peak pressure
VOR	Vestibulo-ocular reflex
VORg	Vestibulo-ocular reflex gain
